# Multimodal Material Classification Using Visual Attention

**DOI:** 10.3390/s24237664

**Published:** 2024-11-29

**Authors:** Mohadeseh Maleki, Ghazal Rouhafzay, Ana-Maria Cretu

**Affiliations:** 1 Department of Computer Science and Engineering, Université du Québec en Outaouais, Gatineau, QC J8X 3X7, Canada; ana-maria.cretu@uqo.ca; 2 Department of Computer Science, Université du Moncton, Moncton, NB E1A 3E9, Canada; ghazal.rouhafzay@umoncton.ca

**Keywords:** material classification, neural objects, visual attention, multimodal sensing

## Abstract

The material of an object is an inherent property that can be perceived through various sensory modalities, yet the integration of multisensory information substantially improves the accuracy of these perceptions. For example, differentiating between a ceramic and a plastic cup with similar visual properties may be difficult when relying solely on visual cues. However, the integration of touch and audio feedback when interacting with these objects can significantly clarify these distinctions. Similarly, combining audio and touch exploration with visual guidance can optimize the sensory examination process. In this study, we introduce a multisensory approach for categorizing object materials by integrating visual, audio, and touch perceptions. The main contribution of this paper is the exploration of a computational model of visual attention that directs the sampling of touch and audio data. We conducted experiments using a subset of 63 household objects from a publicly available dataset, the ObjectFolder dataset. Our findings indicate that incorporating a visual attention model enhances the ability to generalize material classifications to new objects and achieves superior performance compared to a baseline approach, where data are gathered through random interactions with an object’s surface.

## 1. Introduction

In everyday life, people frequently encounter objects that, although visually similar, are composed of different materials. For example, decorative artificial fruits and vegetables can closely mimic their real counterparts. Human cognition leverages multiple sensory channels that work together, enabling us to recognize and identify the materials of objects in our surroundings.

In the AI era, where the goal of reproducing human intelligence extends across various tasks, it is crucial to develop integrated perceptual intelligence. This advancement will enable robots to categorize the diverse array of objects in their environments and accurately identify their materials. This capability is essential for reproducing human ability to recognize and differentiate objects that may visually appear similar. A practical application of this technology could include service robots sorting objects by material—such as paper, plastic, and metal—into recycling bins, enhancing efficiency and accuracy in waste management. Beyond waste handling, these robots could also revolutionize manufacturing and assembly processes by precisely categorizing components based on their material composition, ensuring streamlined production and high-quality outputs. In the construction industry, such robots could optimize the sorting and management of construction materials like bricks, concrete, and metals on-site, improving inventory control and reducing waste. Moreover, in healthcare settings, robots equipped with advanced material recognition capabilities could assist in organizing medical supplies and equipment, ensuring quick access to sterile plastics, metal instruments, and disposable items, thereby enhancing operational efficiency and patient care.

To enable robots to efficiently recognize and differentiate objects, they need to capitalize on data coming from multiple sensory sources. Meaningful solutions need to be proposed to jointly use such data to improve the decision-making process. One such solution is multisensory fusion of information. It can occur at the data level, feature level, or decision level [1]. Alternatively, drawing inspiration from the sensory processing in humans, some types of sensory data, for example, data coming from vision, can guide the acquisition of other types of sensory data when additional information is required to classify the object. The latter approach is used in this study.

In particular, we incorporate visual cues as a guide to selectively collect touch and audio data. We investigate the integration of visual information through a computational model of visual attention. This model examines the surface of an object and outputs its most notable visual features, known as saliencies. Previous research has demonstrated the efficacy of visual cues in directing the touch image sampling process for object recognition via touch [2]. Building on this foundation, our work explores this selective data sampling strategy and applies it to visual, touch, and audio sensory channels, aiming to enhance material recognition. As such, a model of visual attention incorporates visual characteristics of objects, including contrast, color opponency in RGB and DKL color spaces, curvature, edge, entropy, intensity, and symmetry [3]. These features are combined to highlight salient regions as bright areas on a black background. Given that the model of visual attention functions on images, we collect images over a 3D object by positioning the camera at the best viewpoints, determined as viewpoints maximizing the number of visible interest points. The 2D maps of salient regions are then projected back onto the 3D space, providing the 3D locations of salient features on the object. Audio and touch information are subsequently collected at these points of interest.

To test and validate our proposed approach, we conducted experiments using 63 objects from the ObjectFolder dataset [4], a sampling of 3D objects that contain a multisensory profile, including visual, touch, and audio feedback when forces are applied at different locations over their surface. This dataset utilizes implicit neural representations to model each object and its properties and thus allows us to prove the concept and experiment with various testing scenarios without requiring the synchronous sampling of real multisensory data. This is a very useful and practical exploration step to allow us to conduct later real experiments. At this stage of the work, by inputting extrinsic camera parameters into the neural-based model, we can generate images of an object from various viewpoints. Also, the accompanying software allows for simulating the sound of objects being impacted at different locations with different force magnitudes. Finally, touch profiles of objects can also be obtained using the TACTO [5] simulation system that creates an imprint of those captured by a DIGIT sensor [6] upon inputting the touch contact point. Each object is categorized as belonging to one of six material types: ceramic, wood, plastic, iron, polycarbonate, and steel. Our goal is to accurately predict the material type of each target object.

For the vision modality, we customized and fine-tuned a ResNet-18 [7] network, pretrained on ImageNet [8], to process RGB images of the objects and predict their material labels. Similarly, we adapted two other ResNet-18 architectures to recognize object materials from touch and audio. The audio network uses a Mel spectrogram of the impact sound produced by the object as input. For the touch sensory modality, the input takes the form of RGB touch images, which are generated and processed by TouchNet to encode geometric information for each vertex at contact points, as simulated by the DIGIT sensor and TACTO system.

As a baseline for evaluation, we conducted experiments using data collected from randomly selected points on the object surface for touch and audio material recognition and from random viewpoints for the visual material recognition. The results were compared against the proposed scenarios, where the model of visual attention was employed to selectively gather salient data. The main contributions of this paper are as follows: (1) using a computational model of visual attention as base for a data sampling strategy to acquire not only touch data but also audio data over the surface of 3D objects (to the best of our knowledge, it is the first time in the literature that a visual attention model is employed to guide the sampling of audio data for the purpose of object recognition); (2) proposing a novel algorithm to maximize the number of visible interest points on the surface of 3D objects to guide the data sampling procedure; and (3) studying the effectiveness of the model of visual attention in guiding the acquisition of visual, touch, and audio data for 3D object recognition.

The paper is organized as follows: Section 2 reviews the related work on material classification and visual attention research. In Section 3, we present the details of the proposed material classification model, examine them, and apply a selective data sampling strategy across visual, touch, and audio sensory channels to enhance material recognition. Section 4 presents experimental results and a comparison. Finally, the work conducted in this paper is concluded in Section 5.

## 2. Related Work

### 2.1. Material Recognition

Material recognition and characterization is a significant focus in robotics and thus extensively explored by researchers. Proposed solutions generally fall into two main categories: contact-based approaches, where a sensor directly interacts with the object to capture its characteristics for material recognition, and non-contact methods.

Contact-based solutions often leverage touch sensors, some of which mimic human touch. Decherchi et al. [9] employ piezoelectric touch sensors to recognize materials by capitalizing on the variance in mechanical impedances found in different materials. This variability directly affects the output of the piezoelectric transducer, facilitating precise material differentiation. Bhattacharjee et al. [10] propose a material recognition method based on conductive heat transfer from the touch sensor to the object. Their touch sensor design integrates a heating element and a temperature sensor, enabling accurate material identification through a heat conductivity analysis. Vibrotactile sensors, as proposed by Sinopov et al. [11], demonstrate success in material recognition through exploratory scratching behaviors. Optical touch sensors, exemplified by GelSight [12], have been efficiently utilized in material characterization, as demonstrated by Yuan et al. [13]. These sensors capture the reflection pattern of light upon contact with an object’s surface. Yeo et al. [14] suggest utilizing radars for material recognition. Despite radars being traditionally viewed as remote sensing tools, their approach requires immediate contact between the object and the sensor shell for effective material identification. Huang and Wu [15] used a bionic touch sensor to gather vibration data while sliding it over different materials and employ machine learning algorithms for texture recognition. Similarly, a novel EIT-based artificial skin [16] has been developed to detect pressure, position, material type, and temperature, further enhancing touch sensing capabilities for robotics.

Non-contact solutions, in contrast, can determine the material from a distance. These methods primarily utilize remote sensing technologies such as Near Infrared Spectroscopy and thermal imaging. For example, Großmann et al. [17] propose using a broad spectrum of infrared wavelengths to accurately differentiate between a wide variety of materials. This approach relies on emissivity, a property specific to each material, which influences how it emits thermal radiation across different spectrums. Additionally, research by Erickson et al. [18] presents multimodal sensing techniques for material recognition. They leverage near-infrared spectroscopy in combination with close-range high-resolution texture imaging to enable robots to estimate the materials of household objects.

Exploring human perception capabilities for material classification has inspired many researchers. Visual appearance and impact sound offer valuable cues for identifying materials. For instance, Fujisaki et al. [19] studied how humans perceive the material of objects using visual and audio information. In their study, 16 participants rated audiovisual stimuli to determine how they perceived different material categories. In the field of surface material recognition, researchers have explored the integration of acceleration signals with surface images as a means of enhancing classification accuracy. When a user moves a hand-held rigid tool across an object’s surface, the tool’s interaction generates an acceleration signal containing essential information about the surface’s material properties. This signal, combined with surface images in a multimodal approach, provides a comprehensive understanding of surface characteristics and significantly enhances the accuracy of material classification [20]. By integrating multiple sensory modalities, such as touch, visual, and audio perception, researchers are able to gain a more comprehensive understanding of material properties and enhance the accuracy of material recognition systems.

### 2.2. Visual Attention in Object Classification

In recent years, the development of 3D computational models of visual attention has significantly advanced object modeling and recognition. These models are inspired by the human visual exploration of objects. They aim to identify and prioritize the salient features and regions over the surface of 3D objects that are crucial for understanding the structure, function, and significance of these objects. By focusing on these important areas, the accuracy and efficiency of recognizing and classifying objects can be enhanced. The literature on computational models of visual attention encompasses extensive research, originating with the traditional model proposed by Itti et al. [3] for image saliency and later expanded to 3D objects. In recent years, the prominence of deep learning has shifted the focus towards leveraging Convolutional Neural Networks (CNNs) to identify salient regions in 3D objects [21]. Such methods rely on highlighting parts of the 3D object that contribute significantly to the decision-making process of a CNN in object classification.

Visual attention models are deeply rooted in principles of human visual perception, drawing inspiration from the two-stage processing mechanism of the human visual system. In the preattentive parallel stage, the entire visual field is processed simultaneously, enabling the rapid detection of fundamental features such as color, motion, orientation, and size. This is followed by a slower serial attentive stage, where selective attention focuses on specific regions of interest for a detailed analysis, leveraging the center–surround organization of the human receptive field to maximize clarity in central areas. This hierarchical process not only simplifies scene understanding by dividing it into computationally less-demanding tasks but also determines the sequence of fixation points, directing attention to the most salient regions of a scene [3]. Studies such as those by Wolfe and Horowitz [22] further elaborate on the attributes guiding visual attention, categorizing them into undoubted (e.g., color, motion), probable (e.g., luminance polarity, depth cues), and possible (e.g., glossiness, lighting direction) features. Beyond these, psychological research highlights additional properties influencing attention, such as the symmetry of object shapes and contextual relevance [23]. Integrating these biological and psychological insights, computational attention models aim to mimic human-like attention mechanisms by dynamically prioritizing features, aligning cross-modal data, and using attention layers for iterative refinement, thus enabling more effective scene analysis and multimodal feature integration.

Moving beyond mere visual perception, recent research has explored how these visual attention models can serve as guidance to construct effective selective sampling strategies across various domains. Such a strategy is exemplified in several studies that have integrated visual attention mechanisms into diverse applications, ranging from 3D modeling to touch object recognition. For instance, H. Dutagaci et al. [24] focus on evaluating 3D interest point detection techniques using human-generated ground truth data. By employing computational models of visual attention, they assess how accurately these techniques identify points of interest compared to human perception standards. This methodological approach not only validates algorithmic performance but also provides insights into optimizing feature sampling in complex 3D environments. Within the context of touch object recognition, researchers draw inspiration from human haptic exploration by integrating visually interesting points as guides for acquiring touch data. Rouhafzay et al. [25,26] develop an enhanced visual attention model identifying critical regions on object. By leveraging visually attended points, the system demonstrates superior performance in accurately identifying and classifying objects through touch interactions. In summary, these studies collectively demonstrate the significant impact of visual attention models as selective sampling strategies. They enhance computational efficiency and perceptual quality across diverse tasks and open up opportunities for future innovations in interactive systems, robotics, and beyond. Future research endeavors are poised to further refine these models and explore their integration with emerging technologies, promising new breakthroughs in sensory perception and intelligent system design.

## 3. Proposed Approach for 3D Object Material Recognition in Multisensory Data

**Figure 1:**
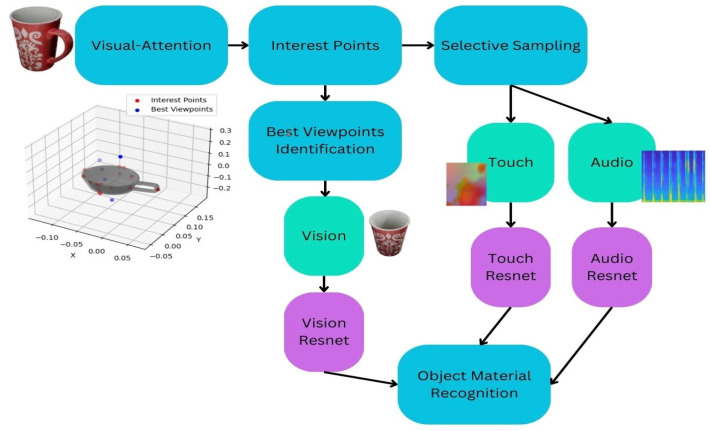
Object material recognition framework.

This section discusses the framework for the proposed approach to material recognition integrating multisensory modalities. As illustrated in Figure 1, our system used multimodal inputs from vision, touch, and audio to accurately identify an object’s material properties. The process began with the application of a visual attention model, where interest points on the object are identified and selectively sampled. Best viewpoints were then determined to capture the most informative visual data (i.e., maximizing the number of visible interest points), which was processed through a vision-based implicit neural network model building upon the ResNet-18 backbone (denoted Vision Resnet) for feature extraction. Simultaneously, touch and audio data were sampled based on the visual attention model. To the best of our knowledge, it was the first time in the literature that a visual attention model was employed to guide the sampling of audio data for object recognition. Resulting touch and audio data were also analyzed using their respective implicit neural network model based on the ResNet-18 architecture, denoted Touch Resnet and Audio ResNet, respectively, in Figure 1. The outputs from these three sensory pathways—vision, touch, and audio—were combined to achieve accurate object material recognition, leveraging the complementary strengths of each modality.

We used a publicly available dataset and software to generate the dataset for our work, namely the ObjectFolder dataset [4]. The objects in this dataset are labeled with six material types: ceramic, wood, plastic, iron, polycarbonate, and steel. Table 1 summarizes the number of objects belonging to each material class. As briefly mentioned above, we trained separate classifiers to recognize objects from various sensory modalities, such as vision, touch, and audio. Our primary objective was to assess the effect of the proposed visually guided data sampling strategy on each modality. To achieve this, we compared the impact of the selective data sampling method with random data sampling.

### 3.1. Data

Using the ObjectFolder dataset [4], which employs implicit neural representations to model each object, we collected 378 RGB images for vision data (6 views per object), 378 touch images, and 378 audio WAV files. With 6 views for each of the 63 objects, this ensured that each object was captured from multiple perspectives. The choice of 6 views per object provided a balanced dataset that allowed the model to learn distinctive visual features from various angles, enhancing its ability to recognize and generalize across different object poses and orientations. To acquire 6 views, we implemented a strategy for random viewpoint sampling by inputting extrinsic camera parameters into the model. This strategy ensured that cameras consistently focus on the center of the object within a specified viewing angle. The camera’s position was defined using spherical coordinates, maintaining a constant radial distance while randomly varying the azimuthal and polar angles. This approach generated images of the object from various viewpoints, ensuring comprehensive visual coverage. Furthermore, the software provided with the ObjectFolder dataset [4] reproduced the sound emitted by the object upon impact at different locations with different force intensities, and the simulated DIGIT sensor replicated the touch properties of the objects, delivering detailed touch feedback aligned with specific touch points. The capabilities of this solution allowed us to validate the work in this paper, as a proof of concept, prior to performing tests with real sensors.

### 3.2. Visual Attention Model for Guiding Visual, Touch, and Audio Data Acquisition for Material Recognition

The proposed solution for guiding data acquisition began with a visual inspection of the object of interest. Using a 3D model of the object and images from multiple views, texture information from the Kinect’s color camera was added to the object’s surface. This enhanced the capabilities of the computational model of visual attention, which incorporated geometrical information (such as edge orientation and curvature) and color properties (such as color opponency and contrast) to identify areas of interest that guide attention.

#### 3.2.1. Features Contributing to the Guidance of Visual Attention

The model of visual attention that we employed integrated various visual characteristics, including intensity, color opponency in RGB, orientation, contrast, curvature, entropy, DKL color spaces, and symmetry [26]. These features were combined to create saliency maps, where salient regions are highlighted as bright areas on a black background. The 2D saliency maps are then projected into 3D space to pinpoint the locations of salient features on the object. The process to compute the visual characteristics can be summarized as follows:

1. Intensity: The first feature analyzed was intensity, which was calculated as the average of the red, green, and blue channels in an image. Since the model operates exclusively on images and we worked on 3D objects, we utilized a series of images taken from different viewpoints of each object. This intensity channel was initially broken down into eight levels using a Gaussian pyramid. Then, center–surround differences were computed by comparing levels 2, 3, and 4 (center) with levels 5, 6, and 7 (surround), respectively. These center–surround operations were inspired by the fact that the human visual system is more sensitive to the center of an image and less to the extremities of the visual field. Each comparison generates two contrast maps (one for intensity increase and one for intensity decrease), resulting in six maps in total. The normalized sum of these six maps produces the final intensity map (denoted as $\;C_{int}$), highlighting regions that stand out based on local intensity contrasts.

2. Color: The human primary visual cortex is thought to respond to red/green and blue/yellow opponency pairs [27]. This concept leads to the division of a visual scene into four broadly tuned color channels, with center–surround differences calculated for the two-color pairs. The color feature map (denoted as ${\;C}_{col}$ in Equation (1)) from [27] was used directly in this work.

3. Orientation: To derive the orientation feature map (denoted as $C_{ori}$), four Gabor pyramids were created for angles of 0°, 45°, 90°, and 135°. Center–surround differences were calculated for each pyramid, and the average of these four maps produced the final orientation feature map, as described in [27].

4. Contrast: Areas with high contrast in a scene tend to draw more attention. In line with this, Harel et al. [28] calculate the contrast map (denoted as ${\;C}_{con}$) in their visual attention model by measuring luminance variations within an 80 × 80 neighborhood. This approach was also used here to incorporate contrast information into the visual attention model.

5. Curvature: Curvature is considered a key factor in directing visual attention [22]. It is notably prominent because it remains unaffected by variations in visual conditions like lighting, shadows, color, or object diffusion properties. Lee et al. [29] derive center–surround differences from the curvature values at each vertex of a 3D model. The same method was employed in this paper to obtain the curvature feature (denoted as $C_{curv}$).

6. Entropy: When working with 3D objects, changes in lighting can lead to the detection of small areas that might not be generally salient. To address this issue, unpredictability in a scene can be quantified as the entropy value [30]. We generated an entropy feature map (denoted as $\;C_{ent}$) by applying a center–surround operation to the entropy values encoded in a 9 × 9 local neighborhood of a median-filtered version of the image.

7. DKL Color Code: Derrington et al. [31] developed a color space where colors are represented in 3D using elevation and azimuth angles. These color space axes reflect the color contrasts of luminance, red/green, and yellow/blue channels based on the color opposition model found in early human vision. In this work, images with RGB color channels were first converted into the DKL color space. Subsequently, spatial decomposition and center–surround operations, similar to those used for previous feature maps, are applied to the color channels obtained (denoted as $C_{DKL}$).

8. Symmetry: Locher et al. [23] observed that the human eye is drawn to the symmetrical axis of a symmetrical object. Building on this observation, we used the algorithm from [32] to identify bilateral and radial symmetric points. These points were marked as bright regions on a dark background, and then, a center–surround operation was applied to produce the final symmetry map (denoted as $C_{sym}$).

#### 3.2.2. Saliency Map Computation and Salient Point Identification

The final saliency map was computed by combining the eight feature maps described in Section 3.2.1 using the following weighted average equation:

(1) $$S_{map}=\frac{w_{col}*C_{col}+w_{con}*C_{con}+w_{curv}*C_{curv}+w_{DKL}*C_{DKL}+w_{ent}*C_{ent}+w_{int}*C_{int}+w_{ori}*C_{ori}+w_{sym}*C_{sym}}{\left.w_{col}+w_{con}+w_{curv}+w_{DKL}+w_{ent}+w_{int}+w_{ori}+w_{sym}\right.}$$

where $C_{col},\;C_{con},\;C_{curv},\;C_{DKL},\;C_{ent},\;C_{int},\;C_{ori}$, and $C_{sym}$ represent the color, contrast, curvature, DKL, entropy, intensity, orientation, and symmetry feature maps, respectively. The weights, $w_{col}\;,\;w_{ori},\;and\;w_{sym},\;w_{con},\;w_{curv},\;w_{DKL}\;,w_{ent},\;w_{int}$, reflect the importance of each feature map. They were calculated based on the Structural Similarity Index method as in [26]. Once these corresponding weights were determined, they were assigned to each conspicuity map to construct the final saliency map. This data fusion method, where different feature maps are combined with specific weights, followed the principle that each feature contributes independently and proportionally to the saliency map. Subsequently, an iterative non-maximum suppression technique [26] was applied to identify the most salient pixels in the saliency map. This technique works by scanning through the saliency map and evaluating each pixel’s value relative to its neighboring pixels. In each iteration, the algorithm retains only the local maxima—pixels that have the highest saliency value compared to their surrounding pixels within a defined window size—while suppressing the values of neighboring, less salient pixels.

#### 3.2.3. Mapping Detected Points from Pixel Coordinates to 3D World Coordinates

Consistent with the methodology outlined in our previous work [2], we employed an orthographic camera projection, wherein all projection lines were parallel. The camera was directed towards the origin, meaning the vector from the camera to the origin was perpendicular to the image plane. Consequently, all vectors from the image plane to their real-world counterparts were also perpendicular to the image plane. We leveraged the ray-intersection algorithm to determine the 3D coordinates of each salient point. In this orthogonal projection, the ray originating from the point on the image plane and aligned with the vector from the camera center to the origin intersected the object at the world coordinates of the salient point.

#### 3.2.4. Maximizing the Number of Visible Interest Points to Identify Optimal Viewpoints

To identify the optimal viewpoints for observing an object, we aimed to maximize the number of visible interest points from each viewpoint. This novel process involved generating a comprehensive set of candidate viewpoints, evaluating their visibility scores, and selecting the most effective ones. Initially, we generated candidate viewpoints by iterating through a range of azimuth angles from 0 to 360 degrees and elevation angles from −90 to 90 degrees. For each combination of angles, we calculated the Cartesian coordinates (x,y,z) based on a predefined distance from the object, ensuring a uniform distribution of viewpoints. These coordinates were stored in a list of candidate viewpoints. Next, we computed the visibility for each candidate by initializing a visibility score to zero and assessing each interest point on the object. A ray was cast from the viewpoint to the interest point, and if it did not intersect with the object mesh, the visibility score was incremented, indicating the interest point was visible. After calculating visibility scores for all viewpoints, we sorted them in descending order to identify the top viewpoints with the highest scores. The top four viewpoints identified through this process were selected, as they provided the most unobstructed views of the interest points.

As mentioned before, our approach utilized the identified salient points for the acquisition of visual, touch, and audio data. For visual data, images were captured by positioning the camera at optimal viewpoints, which were determined by maximizing the number of visible interest points, as shown in Algorithm 1 below. The sampling of optimal viewpoints for touch and audio data in Figure 1 was not directly incorporated into the data sampling process. Instead, the approach involved using interest points, identified through visual attention mechanisms, as the contact points for collecting touch and audio data. Since visual data cannot be captured directly from these contact points, we determined the best viewpoints (to collect visual data) by identifying positions where the number of observed salient points was maximized.
**Algorithm 1:** The Pseudo-Algorithm to Maximize the Number of Visible Interest Points1://1. Generate Candidate Viewpoints2:**for** azimuth_angle in range (0, 360):3:**for** elevation_angle in range (−90, 90):4://Calculate (x, y, z) coordinates based on the distance from the object5:(x, y, z) = calculate_coordinates (azimuth_angle, elevation_angle, distance_from_object)6://Store (x, y, z) in candidate_viewpoints candidate_viewpoints.add ((x, y, z))7://2. Compute Visibility8:**for** viewpoint in candidate_viewpoints:9:visibility_score = 010:**for** interest_point in interest_points:11://Cast a ray from the viewpoint to the interest point12:ray = cast_ray (viewpoint, interest_point)13://Check if the ray intersects with the object mesh14:**if not** ray_intersects_object (ray, object_mesh):15://If no intersection occurs, increment visibility_score16:visibility_score += 117://Store the visibility_score for the viewpoint18:viewpoint_scores[viewpoint] = visibility_score19://3. Find Best Viewpoints20://Sort candidate_viewpoints based on visibility scores21:sorted_viewpoints = sort_by_visibility(viewpoint_scores)22://Select the top 5 viewpoints with the highest visibility scores23:best_viewpoints = sorted_viewpoints [0:5]24://4. Output Best Viewpoints25://Save the top 5 viewpoints to an output file26:save_to_file (best_viewpoints, “output_file.txt”)

After identifying the interest points on the object’s surface, the data acquisition process is simulated at those specific points, by providing those specific points as extrinsic parameters to the ObjectFolder 1.0 software.

We used the process for simulating and rendering images of objects from [4] at specific points identified by the visual attention model. Objects were initially normalized into unit cubes to ensure consistent scaling. For lighting, we used the exact lighting setup described in [4]. Camera viewpoints were initially sampled from a full sphere to capture a range of perspectives, but we then positioned the camera at the optimal viewpoint based on our detection of the best visual angle to ensure the clearest and most informative view of the object. Similarly, for audio simulation, we used linear modal analysis to model impact sounds [4]. This involved converting the object’s surface mesh into a volumetric hexahedron mesh and solving the linear deformation equation to derive vibrational modes. The modes were used to generate audio spectrograms, which were processed by AudioNet to predict complex spectrograms. AudioNet, a neural network designed for sound synthesis, encodes vibration modes from object vertices and predicts the real and imaginary parts of the complex spectrogram using spatial and spectrogram coordinates as input. The mode signals can then be recovered from the spectrogram using the inverse short-time Fourier transform (ISTFT). In [4], the authors used TACTO, a vision-based touch simulator, to generate high-quality touch signals using the DIGIT touch sensor. The sensor makes contact with each object’s vertex along its normal direction, and the resulting RGB tactile images are processed by TouchNet to encode the geometric information of each vertex. In our simulation, we adapted this approach by using points obtained from the visual attention model. These points, which correspond to salient regions on the object’s surface (such as vertices or key features identified by the attention mechanism), were projected onto the 3D model of the object. The 3D coordinates of these contact points were then used as input to TouchNet, allowing us to generate tactile RGB images that captured the geometric information of the object’s surface based on the visual attention mechanism. Importantly, at this proof-of-concept stage of our work, instead of generating sensory data through traditional simulation methods, we directly inputted specific points as extrinsic parameters into VisionNet, AudioNet, and TouchNet, as designed in the ObjectFolder framework.

Using a computational model of visual attention to guide the sampling of visual, touch, and audio data, we collected 252 visual images, 252 touch images, and 252 audio WAV files. Initially, we intended to collect 378 RGB images and 378 audio files, representing 6 views for each of the 63 objects, similar to random sampling. However, with visual-attention-based sampling, the final dataset contained 252 images and audio files, suggesting that some samples may have been omitted or were deemed unusable during the sampling process. Although random sampling resulted in 378 samples across all modalities, which was more than in visual-attention-based sampling, the results from the visual-attention-based approach were superior, as it will be demonstrated in the remainder of the paper.

### 3.3. Proposed Multisensory Classification Method for Vision, Touch and Audio Data

Regarding the scope of this section, we describe the proposed arhcitctures based on machine learning to classify multisensory data. As we further plan to integrate this work in a real sensor system, the focus was towards efficient, fast, and lightweight architectures that are well suited for small datasets and resource-constrained applications.

#### 3.3.1. Vision Data Classification

As briefly mentioned before, we treated each sensory data source separately. For vision, we customized and fine-tuned a ResNet-18 network architecture (denoted as Vision Resnet in Figure 1), pre-trained on ImageNet to analyze RGB images of objects and classify their materials into one of six categories. We chose ResNet-18 as the backbone model for each modality due to its proven performance in image classification tasks and its ability to effectively handle deep architectures without overfitting. ResNet-18 uses residual connections, which allow the network to learn deeper representations while mitigating the problem of vanishing gradients, a common issue in deep networks. With approximately 11.7 million parameters, this makes it an efficient and reliable choice, especially when dealing with relatively smaller datasets like ours. Furthermore, the 18-layer depth of ResNet-18 provides a good balance between performance and computational efficiency, making it an ideal candidate for both feature extraction and multimodal fusion tasks. While other models, such as VGG or deeper ResNet variants like ResNet-50 or ResNet-101, may offer higher classification accuracy, they come with significantly higher computational costs and longer training times. These models might be overkill for our task, especially considering the limited amount of training data available. Additionally, models like DenseNet are known for their dense connectivity, but they tend to have more parameters and require more resources to train, which could lead to slower experimentation cycles. In comparison to these alternatives, ResNet-18 offers an optimal trade-off between efficiency and performance. Given the multimodal nature of our problem, where each modality (vision, touch, and audio) required a separate feature extraction process, ResNet-18’s simple yet effective architecture allowed us to easily adapt it to the three different modalities without introducing significant computational overhead. Additionally, the network’s residual connections enable the model to learn robust features from each modality, which are then effectively fused in subsequent layers to enhance classification accuracy. A detailed comparison of ResNet with other models, such as DenseNet and EfficientNet, is provided in Section 4. DenseNet121 and EfficientNet-B0 exhibit distinct architectural strategies: DenseNet121 incorporates 121 layers, with densely connected convolutional layers ensuring feature reuse and improved gradient flow, while using 4 dense blocks interspersed with transition layers that include pooling operations, resulting in a total of 121 convolutional and pooling layers with approximately 8 million parameters. In contrast, EfficientNet-B0 employs a compound scaling technique, optimizing its depth, width, and resolution, and consists of 18 convolutional and pooling layers with significantly fewer parameters (5.3 million). In this work, we utilized both DenseNet121 and EfficientNet-B0 to evaluate and compare their performance.

As part of the preprocessing setup, we used the original 256 × 256 images without resizing, ensuring that the native resolution was maintained. We then center cropped the images to 224 × 224 pixels. This cropping standardized the input dimensions for the ResNet-18 network, which expected this specific size, while preserving the critical central portion of each image. By focusing on the most relevant area, the model could consistently learn and recognize important features, minimizing the influence of less informative edge regions. Additionally, we normalized the data using a mean of [0.485, 0.456, 0.406] and a standard deviation of [0.229, 0.224, 0.225]. These values represent the mean and standard deviation of pixel intensities in the ImageNet dataset, which is commonly used for training large-scale vision models. It is important to maintain the same mean and standard deviation normalization during fine-tuning or evaluation to ensure consistency with the training process. Data augmentation techniques were applied to the training set to improve the model’s robustness. These techniques included random horizontal and vertical flips, random rotations of up to 15 degrees, color jitter, Gaussian blur with a kernel size of 3, and random grayscale conversion with a probability of 0.1.

The custom neural network architecture integrated a pre-trained ResNet-18 model with a modified fully connected layer tailored to the material classification task. Specifically, the fully connected layer was adapted to classify images into different material categories. During training, we initialized model parameters, utilized a Cross Entropy Loss function, and optimized using the Adam optimizer with a learning rate of 0.0001, betas of (0.9, 0.999), an epsilon of 1e-8, and weight decay of 0. The training process included a learning rate scheduler that decayed the learning rate by a factor of 0.1 every 7 epochs. The results are presented in Section 4.

#### 3.3.2. Touch Data Classification

For the touch model, denoted as Touch Resnet in Figure 1, we also utilized a pre-trained ResNet-18 architecture. This architecture was employed to process and analyze the RGB touch images generated by the TACTO simulator, which represented the local contact geometry at the salient points. We constructed a custom dataset class to manage the loading and preprocessing of images from the touch dataset. The training loop includes updating the learning rate periodically to ensure efficient convergence. The dataset was loaded using a DataLoader with a weighted random sampler to balance the class distributions. Similar to the vision model, we fine-tuned a ResNet-18 architecture pre-trained on ImageNet, modifying the final fully connected layer to output the correct number of classes for our dataset. The model was trained using Stochastic Gradient Descent (SGD) optimizer with a learning rate of 0.001 and a momentum of 0.9 [33]. We experimented with both SGD and Adam optimizers, tuning their hyperparameters to identify the best fit for our model and data. The results of these experiments demonstrated that SGD performed better, leading to more stable and consistent convergence for our specific task. A learning rate scheduler with a step size of 7 and a gamma of 0.1 was employed to adjust the learning rate during training. To prevent overfitting, an early stopping mechanism was implemented. The model’s training and validation losses and accuracies were plotted over epochs (see Section 4) to visualize the learning progress and effectiveness.

#### 3.3.3. Audio Data Classification

For processing audio data and training an audio-based material classifier, we developed a custom dataset class to manage the loading and preprocessing of the audio files. This class enabled efficient handling of the entire audio dataset and ensured the data were structured for use with the classifier. Each audio file underwent transformations, including Mel-Spectrogram conversion, time masking, and frequency masking, to enhance the model’s robustness. We selected Mel-Spectrograms for audio data processing because they efficiently represented the frequency content of audio signals in a way that aligned well with how human hearing perceives sound. Mel-Spectrograms convert audio files into a time–frequency representation, which captures both the amplitude and frequency information across time. This transformation helped to highlight the relevant features of the audio data, making it easier for the classifier to learn and differentiate between different types of materials based on their acoustic characteristics. Each audio file underwent this Mel-Spectrogram conversion to ensure that the data fed into the model were consistent and optimized for accurate classification. We employed a modified ResNet-18 architecture, denoted Audio Resnet in Figure 1, tailored for audio data by adjusting the first convolutional layer for single-channel input. We also plotted training and validation loss and accuracy over epochs (see Section 4) to monitor the model’s performance, providing insights into the model’s learning process and generalization capabilities.

#### 3.3.4. Vision + Touch + Audio Data Classification

We enhanced the model architecture by utilizing distinct ResNet-18 models to classify data from three different modalities: vision, touch, and audio. Each modality had its own dedicated ResNet-18 backbone, which extracted modality-specific features. By adapting ResNet-18 for feature extraction across all modalities, we simplified the fusion of these diverse inputs by concatenating the extracted feature vectors into a unified representation. The fusion process involved concatenating the flattened feature vectors from the ResNet-18 backbone of each modality, creating a single unified feature vector. This concatenation happened at the feature level, where we combined the outputs from the final layers of each ResNet-18 model. After concatenation, the combined feature vector iwass passed through a fully connected Multilayer Perceptron (MLP) that was responsible for performing the multimodal fusion and classification. The MLP is designed to learn the complementary relationships between the different modality features, thus improving accuracy and generalization across multimodal datasets. This allowed for a streamlined yet robust feature extraction process. After concatenation, the combined feature vector was passed through a fully connected Multilayer Perceptron (MLP) designed for multimodal fusion and classification. This approach capitalized on the proven feature extraction capabilities of ResNet-18 while introducing efficiency by training a shared fusion model for all modalities. By learning complementary features from each modality, the model not only reduced computational overhead but also improved accuracy and generalization across multimodal datasets, effectively recognizing materials through vision, touch, and audio data (see Section 4 for the corresponding results).

## 4. Evaluation

As stated before, we conducted an evaluation process employing two sampling strategies, random sampling vs. visual attention sampling, across three sensory modalities: vision, touch, and audio. Specifically, the paper argues that employing a model of visual attention as a data sampling strategy enhances the likelihood of recognizing objects across the three modalities. To validate this, we compare the performance of trained networks on test samples selected through visual-attention-driven sampling against a baseline of randomly selected samples. As detailed in Table 2, which shows the performance of models on each modality, training on visual data with random sampling yielded an average accuracy of 66.1%. Employing visual attention during training resulted in a notable improvement, with the model achieving a material classification accuracy of 81.5%. Similar trends were observed with touch data, where visual attention demonstrated approximately a 3% increase in performance compared to random initialization. Remarkably, in the case of audio data, which exhibited the highest accuracy for material recognition, employing visual attention led to even further enhancements in accuracy.

These findings demonstrate the significant improvement in accuracy achieved by incorporating visual attention mechanisms compared to random sampling. Although the random sampling method utilized 378 samples and the visual attention method employed only 252 samples, with both training and test datasets being smaller for visual attention, the results demonstrated superior performance for the visual attention approach. This indicates that visual attention mechanisms can enhance model accuracy more effectively than random sampling, even with a reduced number of samples.

### 4.1. Computational Efficiency and Model Comparison

To evaluate the effectiveness of ResNet-18 as the base model, we compared its performance with DenseNet and EfficientNet across all modalities (vision, touch, and audio), using both random sampling and visual attention sampling strategies. Table 3 summarizes the accuracy and training/testing times achieved by each model under different configurations. The experiments were all run on a GPU system. Specifically, an NVIDIA Tesla T4 with 15.36 GB of VRAM and CUDA Version 12.2 was used. Leveraging GPUs, especially for the training phase, offers a significant reduction in processing time, which is particularly beneficial for real-time applications. The pretrained network can then be deployed, depending on the specific need, on resource-constrained platforms, such as edge devices or embedded systems to perform real-time inference.

The results demonstrate that DenseNet achieved higher accuracy than ResNet-18 for vision modality, particularly with visual attention sampling, but at the cost of increased computational complexity. Similarly, EfficientNet showed competitive performance but struggled in audio classification under visual attention. For tactile data, ResNet-18 required 649 s (~11 min) for training and 1–2 s for testing, compared to DenseNet’s 1135 s (~19 min) and 2 s for testing and EfficientNet’s 655 s (~11 min) and 1 s for testing. Similar trends were observed across vision and audio modalities, where ResNet-18 consistently offered faster processing times while maintaining reasonable accuracy. These findings highlight ResNet-18’s lightweight architecture and efficiency, making it well suited for small datasets and resource-constrained applications. As such, ResNet-18 was chosen as the backbone model for the remainder of this work.

### 4.2. Confusion Matrix Analysis for Performance Evaluation

To further validate the performance of the ResNet-18 model, we analyzed the confusion matrices for each modality using both sampling strategies to obtain insights into specific misclassifications and on the impact of visual attention mechanisms on model performance. While the overall accuracy varied across modalities, the confusion matrix analysis helped us understand the underlying reasons for these differences, especially in terms of misclassifications that contributed to the accuracy disparities between modalities.

**Figure 2:**
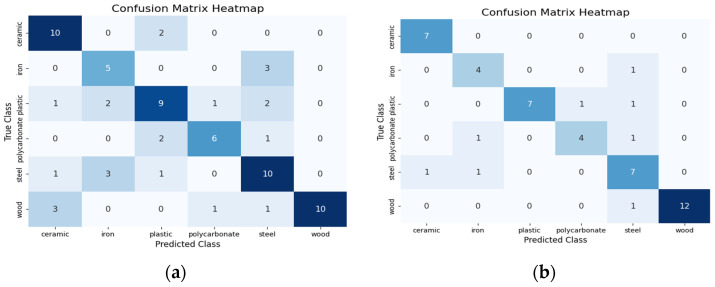
Confusion matrices based on visual data modality (**a**) for random sampling and (**b**) for visual attention sampling.

For the vision modality, errors observed with random sampling were concentrated in classes with similar visual properties. For example, plastic was misclassified as iron in two cases, as steel in another two cases, as ceramic in one case and as polycarbonate in one case. Overall, there are a total of 24 misclassifications over all materials types when using random sampling, as shown in the confusion matrix in Figure 2a. However, with visual attention sampling, the total misclassifications over all material types were reduced to eight. Notably, confusion between plastic and iron was eliminated, and the misclassifications of plastic as steel decreased to one, as illustrated in Figure 2b. This demonstrates the attention mechanism’s ability to focus on distinctive visual patterns, significantly reducing errors.

**Figure 3:**
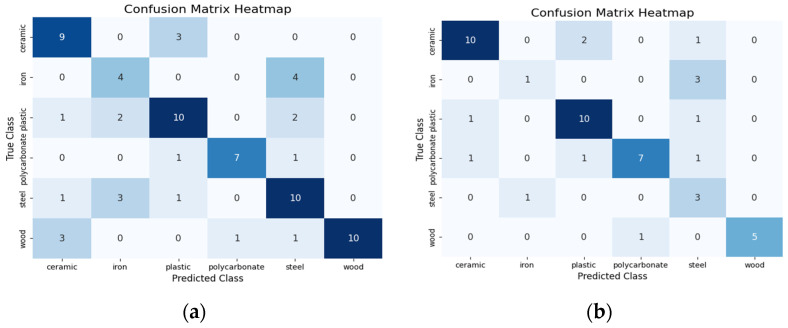
Confusion matrices based on touch modality (**a**) for random sampling and (**b**) for visual attention sampling.

For the touch modality, random sampling misclassifications were primarily observed among tactilely similar materials, such as iron and steel or plastic and polycarbonate, resulting in a total of 24 misclassifications. The confusion matrix in Figure 3a highlights these trends, with plastic frequently misclassified as steel in two cases and as iron in two cases. Visual attention sampling significantly reduced these errors to 13 (a total of 14 misclassifications), as shown in Figure 3b. For instance, misclassifications of plastic as steel decreased from two cases to one. This improvement demonstrates the model’s ability to better leverage tactile distinctions when guided by attention mechanisms. However, the touch modality still faced challenges in fine-grained material differentiation, indicating the potential for improvement by integrating more detailed tactile features or expanding the dataset.

**Figure 4:**
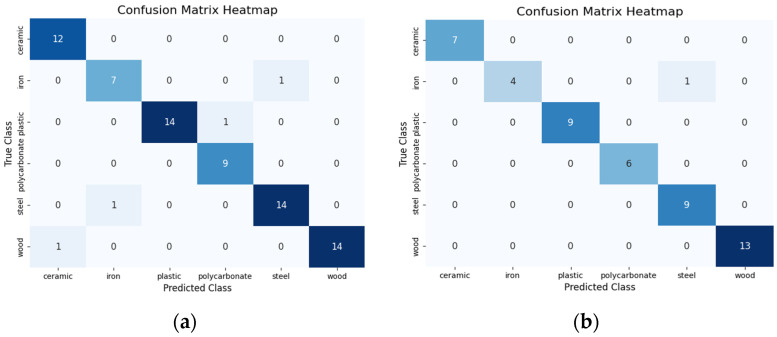
Confusion matrices based on audio modality (**a**) for random sampling and (**b**) for visual attention sampling.

For the audio modality, random sampling resulted in minimal errors, with most misclassifications involving confusion between iron and steel, plastic and polycarbonate, and ceramic and wood, one misclassification for each, leading to a total of four classification errors. The confusion matrix in Figure 4a highlights this, with one instance of iron being misclassified as steel and vice versa. Visual attention sampling further reduced these errors to just one, as shown in Figure 4b. This demonstrates the effectiveness of visual attention in reinforcing the distinctiveness of acoustic features for material recognition. Despite the overall strong performance, subtle acoustic similarities between certain materials, particularly iron and steel, remain challenging, suggesting the need for further refinement of audio feature extraction techniques.

The confusion matrix analysis also revealed a potential synergy across modalities: while vision and touch modalities struggled with differentiating between iron and steel, the audio modality consistently showed better performance in distinguishing these materials. This suggests that a multimodal approach combining vision, touch, and audio can achieve near-perfect classification by leveraging their complementary strengths. Multimodal fusion can thus improve the classification accuracy by resolving misclassifications between similar materials, particularly where one modality performs well but another one struggles.

Furthermore, the confusion matrices highlight how visual attention not only improves accuracy but also addresses specific challenges in distinguishing visually and tactically similar materials. Despite the improvements from visual attention, certain materials, like plastic and polycarbonate, remain difficult to classify accurately across all modalities. This highlights a limitation in feature extraction and suggests that future work could focus on fine-tuning the model’s ability to capture more detailed features. Additionally, the smaller sample size used for visual attention sampling (252 samples) may have limited the model’s generalization capabilities in some cases, suggesting the need for larger, more diverse datasets to further improve performance.

### 4.3. Leveraging Majority Voting for Robust Model Performance Assessment Across Modalities

The evaluation process in this work also employs an optimized method that leverages majority voting to assess the model’s performance using different combinations of test data samples. Recognizing objects from a single touch image can be challenging, so we conduct experiments using multiple touch images and maintain consistency across modalities by applying the same approach to all. As such, we selected combinations of test samples in sizes of 1, 3, and 5, generating random combinations for each sample while ensuring the sample itself was always included. The model was applied to these combinations, and majority voting was used to determine the final prediction for each sample, which was then compared to the true labels. This approach was applied to both the random and visual-attention-based sampling strategies. Additionally, we generated sets of three or five images (or Mel-Spectrograms) from the test data and calculated the model’s accuracy for each sample size, as shown in Table 4 and Table 5. By determining the optimal number of samples for accurate material identification, we aim to enhance results and provide a more robust assessment of the model’s performance.

As illustrated in Table 4, Table 5 and Table 6, transitioning from one to three samples notably improved accuracy, with additional improvements evident when moving to five samples. In fact, for single samples, the model’s performance is the same as its normal performance on individual test data. In this case, there is no combination or majority voting, and the result is directly derived from the model’s prediction on that single sample. Therefore, this performance should be similar to the model’s overall performance on the entire test data. However, when larger combinations (e.g., three or five samples) are used, the goal is to determine if majority voting over multiple samples can provide better results compared to a single sample. Visual attention consistently outperformed random sampling across all sensory modalities, particularly in scenarios with a higher number of samples, suggesting its efficacy in filtering out noise and focusing on pertinent features, thereby facilitating more reliable decision-making.

As illustrated in Table 6, where we combine all three modalities—vision, touch, and audio—we observe a marked improvement in classification accuracy for both the vision and touch modalities. However, the performance of the audio modality remains comparable to its previous results, showing only a slight increase.

**Figure 5:**
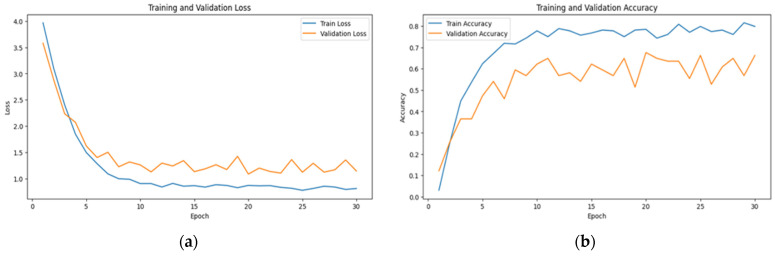
The learning curve showing (**a**) the loss and (**b**) the accuracy for visual data with random sampling.

**Figure 6:**
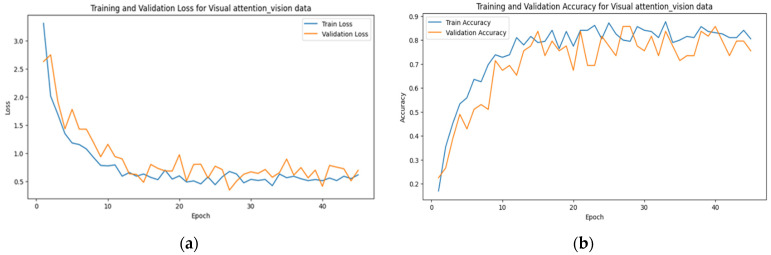
The learning curve showing (**a**) the loss and (**b**) the accuracy for visual data with visual attention sampling.

The learning curves of multimodal systems shown in Figure 5 and Figure 6 illustrate that the performance benefits associated with visual attention become more pronounced over time. This suggests that as the system learns to discern patterns and prioritize relevant information, the advantages of visual attention mechanisms become increasingly apparent.

**Figure 7:**
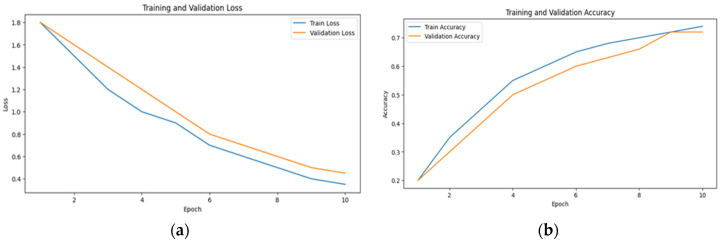
The learning curve showing (**a**) the loss and (**b**) the accuracy for touch data with visual attention sampling.

**Figure 8:**
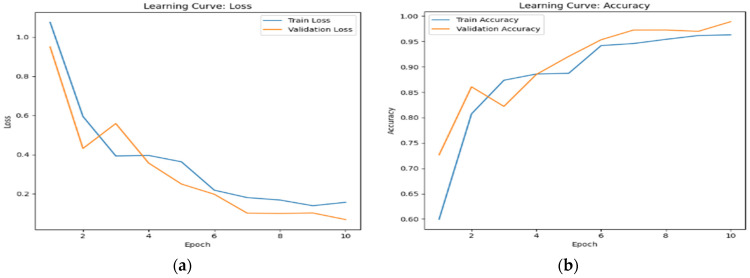
The learning curve showing (**a**) the loss and (**b**) the accuracy for audio data with visual attention sampling.

**Figure 9:**
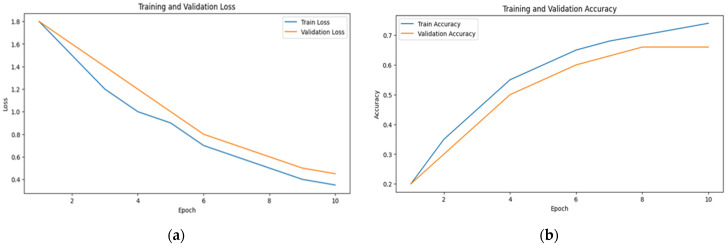
The learning curve showing (**a**) the loss and (**b**) the accuracy for touch data with random sampling.

**Figure 10:**
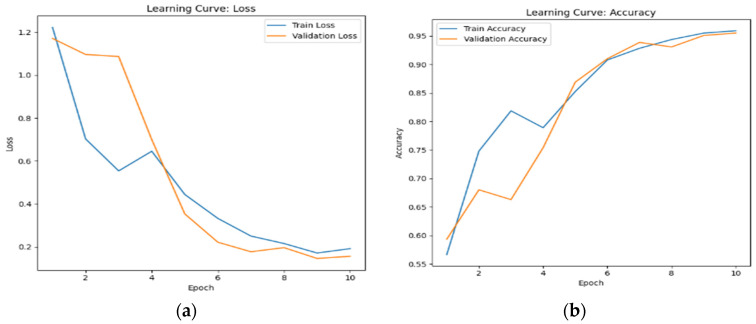
The learning curve showing (**a**) the loss and (**b**) the accuracy for audio data with random sampling.

This fact is reflected in the steady increase in accuracy and the decrease in loss over time, as observed in Figure 6, Figure 7 and Figure 8. The scalability of visual attention is also demonstrated by the model’s continuous improvement and adaptability to new data, which is evident in the consistent progression of the learning curves in Figure 6, Figure 7 and Figure 8 compared to Figure 5, Figure 9 and Figure 10. This adaptability allows the system to continuously improve as it encounters new data.

## 5. Conclusions

In this work, we demonstrated the effectiveness of using a computational model of visual attention to guide the sampling of visual, touch, and audio data in order to enhance material recognition over the surface of 3D objects. By focusing on salient features of objects, the model enables more efficient and accurate data sampling compared to random sampling. Our proof-of-concept experiments using the ObjectFolder dataset show that this selective approach leads to improved material classification across various sensory modalities. The results highlight the potential of integrating multiple sensory channels to better understand and recognize materials, with significant implications for applications in robotics, manufacturing, and material science. The fine-tuning of ResNet-18 networks for each modality—visual, touch, and audio—showed that targeted data sampling based on visual saliencies provides a superior strategy for recognizing material types like ceramic, wood, plastic, iron, polycarbonate, and steel. While DenseNet demonstrated slightly higher accuracy for some modalities, particularly in visual tasks, and EfficientNet offered competitive performance, ResNet-18 was ultimately chosen for its balance of accuracy, computational efficiency, and simplicity in practical applications. Overall, our approach illustrates the benefits of leveraging visual attention models for multisensory data integration, paving the way for more advanced and perceptive material recognition systems.

Future research endeavors will delve into the specific mechanisms underlying visual attention and its impact on decision-making processes within multimodal systems. Understanding how attentional mechanisms prioritize sensory inputs and guide information processing could pave the way for the development of more sophisticated algorithms and models. In our current study, we use random sampling as a baseline technique to demonstrate the potential of visual-attention-based sampling in the context of multimodal data. Additional improvements to the visual attention model, such as the addition of curvature and Laplacian values for mesh vertices, will be implemented in future work to explore alternative methods for selecting salient points from touch and audio data to further validate the proposed method. Once the theoretical approach is thoroughly tested and validated, future research will be dedicated to testing our approach with real sensors that will allow us to study more objects and more material types.

## Figures and Tables

**Table 1:** The material distribution of the 63 objects used in experiments. (# represents the number of objects).

Material	#Number of Objects
Ceramic	14
Plastic	13
Polycarbonate	10
Steel	10
Wood	10
Iron	6

**Table 2:** Accuracy (in %) across different sensory modalities for both random and visual attention sampling.

	Vision	Touch	Audio
Random sampling	66.1	66.38	94.87
Visual attention sampling	81.5	74.21	98.91

**Table 3:** Comparison of model performance (ACC, accuracy in %) and computational efficiency (training and testing time, computed using GPU) for ResNet-18, DenseNet121, and EfficientNet-B0 for vision, touch, and audio modalities using both visual attention and random sampling strategies.

Modality	Sampling Strategy	ResNet-18 ACC	DenseNet ACC	EfficientNet ACC	ResNet-18 Train Time (s)	ResNet-18 Test Time (s)	Dense Net Train Time (s)	Dense Net Test Time (s)	EfficientNet Train Time (s)	EfficientNet Test Time (s)
Vision	Visual Attention	81.50	92.07	89.01	1401	6–7	2105	7	1565	7
Vision	Random	66.10	88.38	76.38	1520	6–7	2578	9	1975	8
Touch	Visual Attention	74.21	65.48	71.26	649	1–2	1135	2	655	1
Touch	Random	66.38	61.04	69.12	976	2–3	1646	5	992	1–2
Audio	Visual Attention	98.91	97.61	94.61	2587	9–10	3206	10–11	2100	9–10
Audio	Random	94.87	88.88	90.18	2731	11–12	3710	12–13	2501	11–12

**Table 4:** Material recognition accuracy for different modalities and numbers of samples per category using random sampling.

Number of Samples	Vision	Touch	Audio
1 (no voting)	70.27	66.33	94.87
3	86.49	71.75	95.80
5	87.84	76.67	97.22

**Table 5:** Material recognition accuracy for different modalities and numbers of samples per category using the proposed visual attention sampling.

Number of Samples	Vision	Touch	Audio
1 (no voting)	81.63	74.21	98.91
3	91.84	84.65	99.20
5	91.84	88.33	100

**Table 6:** Voting with multimodal fused network.

Number of Samples	Vision + Touch + Audio
1 (no voting)	98.92
3	99.58
5	100

## Data Availability

We use the public dataset ObjectFolder 1.0, which is openly available in the ObjectFolder repository at https://github.com/rhgao/ObjectFolder (accessed on 27 November 2024) or via the dataset’s DOI/URL [https://download.cs.stanford.edu/viscam/ObjectFolder/ObjectFolder1.0.tar.gz (accessed on 27 November 2024)].
